# Enhanced levels of double-strand DNA break repair proteins protect ovarian cancer cells against genotoxic stress-induced apoptosis

**DOI:** 10.1186/1757-2215-6-66

**Published:** 2013-09-17

**Authors:** Rajkumar Singh Kalra, Sharmila A Bapat

**Affiliations:** 1 National Centre for Cell Science, NCCS Complex, Pune University Campus, Ganeshkhind, Pune 411 007, India

**Keywords:** SeOvCa progression model, DSB-repair, HR and NHEJ pathways, Aneuploidy

## Abstract

**Background:**

Earlier, proteomic profiling of a Serous Ovarian Carcinoma (SeOvCa) progression model in our lab had identified significantly enriched expression of three double-strand break (DSB) -repair proteins *viz.* RAD50, NPM1, and XRCC5 in transformed cells over pre-transformed, non-tumorigenic cells. Analysis of the functional relevance of enhanced levels of these proteins was explored in transformed ovarian cancer cells.

**Methods:**

Expression profiling, validation and quantitation of the DSB-repair proteins at the transcriptional and protein levels were carried out. Further analyses included identification of their localization, distribution and modulation on exposure to Estradiol (E_2_) and cisplatin. Effects on silencing of each of these under conditions of genomic-stress were studied with respect to apoptosis, alterations in nuclear morphology and DNA fragmentation; besides profiling known mitotic and spindle check-point markers in DSB-repair.

**Results:**

We identified that levels of these DSB-repair proteins were elevated not only in our model, but generally in cancer and are specifically triggered in response to genotoxic stress. Silencing of their expression led to aberrant DSB repair and consequently, p53/p21 mediated apoptosis. Further compromised functionality generated genomic instability.

**Conclusions:**

Present study elucidates a functional relevance of NPM1, RAD50 and XRCC5 DSB-repair proteins towards ensuring survival and evasion of apoptosis during ovarian transformation, emphasizing their contribution and association with disease progression in high-grade SeOvCa.

## Background

Expression of numerous markers associated with DNA damage signaling and repair pathways have been reported in malignant cells [1]. It has also been suggested that such proteins are expressed in response to several genotoxic therapies including radiation and chemotherapeutic drugs [2]. DNA double-strand break (DSB) repair, more specifically homologous recombination (HR) mediated repair pathway is reported to be frequently disrupted in solid high-grade serous ovarian adenocarcinoma (SeOvCa) tumors [3]. Overexpression of such DSB-repair associated proteins is assumed to be associated with endogenous replication stress and high frequency of DNA breaks in transformed cells [4,5], though functional relevance of these markers remained largely uncharacterized during the disease progression.

The basis of the current study is protein expression profiling of SeOvCa progression model that was carried out earlier and identified differential protein patterns [6]. This protein profiling study was carried out using an *in vitro* model of SeOvCa progression established earlier in our lab [6,7]. In brief, several single cell clones were isolated from the malignant ascites of a grade IV SeOvCa patient. During subsequent culture, nineteen of these clones underwent spontaneous immortalization. One of these immortal clones *viz*. A4 with slow-cycling and non-tumorigenic properties, got transformed (passage ~20-25) into an aggressively proliferating clone that exhibited tumorigenic and metastatic capabilities in *in vitro* assays. This constitutes the SeOvCa progression model system, wherein early immortal A4 cells with non-tumorigenic potential were termed as pre-transformed or A4-P, and transformed A4-P derived tumorigenic and metastatic cells were termed as A4-T (Additional file 1). With distinct cellular phenotype, such isogenic cellular system provided a suitable progression model of two functionally discrete cell types derived from a single clone. Protein profiling of the progression model led to derivation of two groups based on their qualitative and differential expression patterns. *Group I* comprised of proteins, qualitatively expressed in either A4-P or A4-T (termed as EEx and LEx proteins based on their identification in Early and Late passage A4 cells respectively), while *Group II* comprised of proteins expressed at quantitatively different levels in each cell types (threshold = > 2, fold change). Categorization of proteins into functional networks provided a clear insight of cellular functionality and major pathways regulating ovarian cell transformation.

Functional annotation of the 34 LEx proteins revealed an association of diverse cellular processes including resistance to apoptosis, energy metabolism, cell proliferation, angiogenesis and invasion and metastases. Broadly these represent some of the classical hallmarks of cancer [8]. One of the progression-associated function was that of DSB-repair in which, three proteins *viz.* RAD50, NPM1 & XRCC5 were identified as being highly enriched in transformed cells. Around 50% of high grade SeOvCa tumors exhibit aberrant DSB-repair functions and high prevalence of mutations in DSB-repair genes [3]. In exploring the functional relevance associated with disease progression, we focused on these three LEx proteins of DSB-repair pathways. NPM1 has been suggested to involved in the process of DNA repair in UV irradiated cells [9] while, loss of NPM1 is found to constitutively activate DNA damage response with increased levels of histone H2AX phosphorylation [10]. NPM1 has been recently identified to be recruited by ubiquitin conjugates downstream of RNF8 and RNF168 in HR pathway [11]. RAD50 as a key component of MRN (MRE11, RAD50 & NBS1) complex participates in both DSB repair pathways of HR and non-homologous end-joining (NHEJ), while XRCC5 is one of the key protein involved in NHEJ repair [12-15].

On this background, to elucidate their contribution to disease progression, we characterized the functional involvement of NPM1, RAD50 and XRRC5 DSB-repair proteins in ovarian cancer. Our study demonstrates an approach of transformed cells towards combating DSB-repair, impairment of which leads to apoptosis and genomic instability.

## Methods

### Cell culture, treatments and transfections

Derivation of the A4 progression model of pre-transformed and transformed SeOvCa cells (A4-P and A4-T cells) is described earlier [7,16]. A4-P cells (between passage numbers 15–17) and A4-T cells (between passage numbers 37–38) were used in the study and cultured stringently to avoid the risk of cross-contamination. The A4-T cells were treated with estradiol (E_2;_ 10 nM) for 48 h and expression analysis of c-Myc, NPM1 and RAD50 was performed. The siRNA pools of negative control, NPM1, RAD50 and XRCC5 (MISSION siRNA; Sigma Aldrich Inc.) were used for generating transient knockdown cells. The A4-T cells were treated with 4 μM cisplatin (Sigma Aldrich Inc.) for 24 h, 48 h time-points and were subsequently used for expression analysis and validation studies. In siRNA transfections, 10 nmol siRNA duplexes were transfected into A4-T cells with Lipofectamine RNAiMax (Invitrogen) according to the manufacturer’s instructions, and cells were analyzed for expression validation after 48 h of transfection. To induce DNA damage and to examine H2AX-γ and NPM1 translocalization, A4-T cells 24 h after transfection were treated with 4 μM cisplatin for 24 h before immunofluorescence analysis. The 4 μM cisplatin treatment for 48 h post 24 h transfection was given to validate protein expression levels of ATM, pATR(Str428), RAD50, NPM1, XRCC5, p53, p21, CDK1, Cyclin D1, Bcl-2 and β-actin.

### Semi-quantitative reverse transcription-PCR

Trizol™ reagent (Invitrogen, USA) was used to extract total RNA from cells as per manufacturer’s guidelines [17]. Semi-quantitative reverse transcription-PCR was performed under standard conditions as described earlier [18] and amplified products were resolved on a 1.5% Agarose gel; β-actin was used as internal control. Gel was run and captured under gel documentation system (Syngene; Cambridge, UK).

### Antibodies, immunoblotting and quantitative analysis

Immunoblotting (IB) was performed as described earlier [18]. Primary antibodies were used at following concentrations for IB and IF (immunofluorescence) applications; anti-NPM1 (Sigma #WH0004869M1), 1:4000(IB), 1:150(IF); anti-RAD50 (Sigma #R1653), 1:1000(IB), 1:100(IF); anti-XRCC5 (Ku86, B-1) (Santa Cruz # sc-5280), 1:2000(IB), 1:100(IF); anti-c-Myc (Origene # TA100010), 1:1000; anti-Gamma H2AX (phospho Ser139) (Abcan #ab-11174), 1:2000(IB), 1:100(IF); anti-ATM (H-248) (Santa Cruz #sc-7230), 1:1000; anti-phospho-ATR(Ser428) (Cell Signaling #2853), 1:1000(IB); anti-p53 (DO-1) (Santa Cruz # sc-126), 1:1000(IB), 1:100(IF); anti-p21 (BD #556430), 1:1500; anti-Bcl-2 [8] (Santa Cruz # sc-130308), anti-Cyclin D1 (BD #554180), 1:1000(IB); ant-Cdk-1 (Santa Cruz # sc-53219), 1:1000; anti-α-tubulin (Sigma #T5168), 1:5000(IF); ant-γ-tubulin (Sigma #5192), 1:1000(IF); anti-MAD2 (E-17) (Santa Cruz # sc-31790), 1:100(IF), and anti-BUBR1 (N-20) (Santa Cruz # sc-16193), 1:100(IF). Probing with an anti-β-actin antibody (Clone AC-15) (Sigma # A1978), 1:10,000(IB); served as a loading control. Secondary antibodies linked with horseradish peroxidase (HRP) were used as follows: anti-rabbit (1:1500), anti-mouse (1: 1500) procured from Amersham (Pharmacia Biotech, Little Chalfont, UK). Immunoblots were scanned and densitometry for quantitative analysis was performed using a Syngene Gene Genius™ Gel Documentation System (Syngene; Scientific Laboratory Supplies; #http://www.syngene.com). Protein expression values normalized with β-actin were represented as relative expression in percentage.

### Cell cycle and apoptosis assay

Cell cycle analysis of transfected cells was performed with PI (Propidium-Iodide) staining using standard procedure [19]. Sample acquisition and data analysis was performed on FACSCalibur (Becton Dickinson, San Diego, CA, http://www.bdbiosciences.com) using ModFit analytical software. Annexin V–FITC apoptosis assay was performed as described earlier [18] and acquisitions were made on FACSCanto II (Becton Dickinson); DiVa software (Becton Dickinson) was used for data analysis.

### Immunofluorescence staining and In-situ fluorescein cell-death detection (TUNEL) assay

Control siRNA, siNPM1, siRAD50 and siXRCC5 transfected A4-T cells were grown on cover slips for 24 h followed by treatment with cisplatin and E_2_ for 24-48 h wherever indicated. After treatment, media was decanted and wells washed with 1X PBS buffer. Cells were fixed with 4% paraformaldehyde and were kept for 10 min on ice. Immunofluorescence staining was performed as described earlier [19] using NPM1, RAD50, XRCC5, H2AX-γ and p53 antibodies; Hoechst was used for nuclear staining. Images were acquired and analyzed on confocal microscope (Carl Zeiss, Jena, Germany). Quantification of intensity of p53 nuclear foci was carried by measuring the expression intensity across nuclei dimension on Leica LAS_AF analysis platform. Intensity of p53 foci was represented on y-axis, while distance or length of nuclei is shown at x-axis. TUNEL Assay was performed as described earlier [18]. Cells with labeling solutions (Roche) were taken as negative control. Cells were washed thrice and stained with Hoechst for nuclear staining. Stained samples were acquired and analyzed on confocal microscope (Leica, Germany).

### Giemsa staining

Control, NPM1, RAD50 and XRCC5 siRNA transfected A4-T cells were grown in 24 well plate and treated with cisplatin (4 μM) and E_2_ (10 nM) for next 48 h. Upon harvesting, cells were washed twice with chilled 1X phosphate buffer saline (PBS) and then fixed with ice-cold absolute methanol for 20 min at 4°C. Cells were rinsed once with PBS and incubated with Giemsa stain for 30 min at RT. Further, cells were washed twice with 1X PBS and analyzed under Olympus microscopy (Olympus Co., Tokyo, Japan) at 40X magnification.

### Statistical analysis

All experiments were carried out in triplicate; data are expressed as mean ± SEM of three independent experiments. The significance of difference in the mean values was determined using two-tailed Student's *t* test; wherein p < 0.05 considered significant. ANOVA test was performed to compare protein expression between the groups at a significance level of < 0.05. Student-Bonferroni test was used to evaluate sub-comparisons to error rate.

## Results

### Transformation-associated DSB Repair proteins are enriched in ovarian cancer

**Figure 1:**
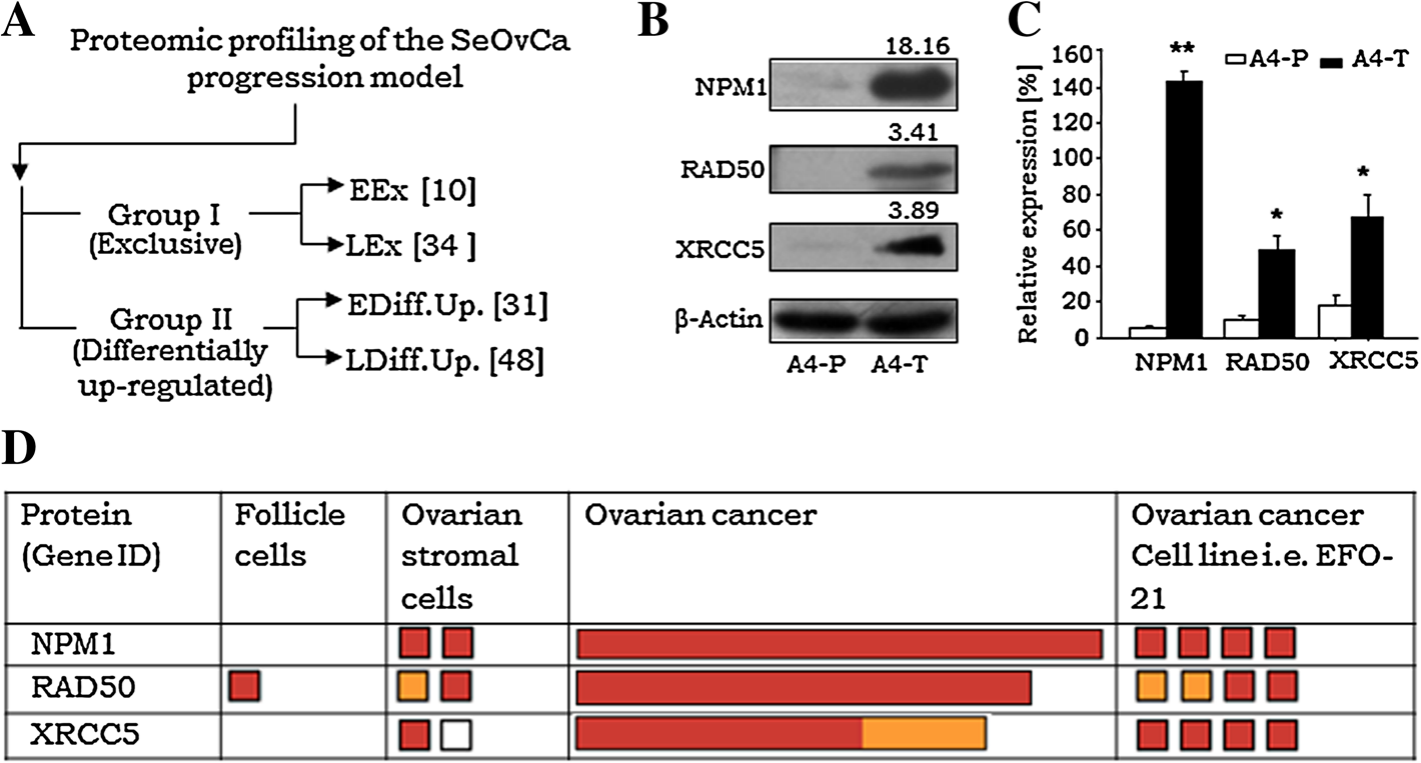
**Validation of NPM1, RAD50 and XRCC5 expression in ovarian cancer. A**. Schematic demonstration showing proteomic profiling of SeOvCa progression model which leads to derivation of two expression groups; *Group I* comprise qualitative expressions, while *Group II* consist of differentially upregulated proteins. (Groups were further divided in two sub-groups of A4-P and A4-T cells). **B**. Representative immunoblot validating qualitative expression of NPM1, RAD50 and XRCC5 proteins in A4-T cells over A4-P cells; numbers indicate mean fold-change values. **C**. Quantitation of NPM1, RAD50 and XRCC5 proteins expression levels. **D**. Expression levels of NPM1, RAD50 and XRCC5 proteins in ovarian cancer samples and cell line (i.e. EF0-21) analyses in HPA database (http://www.proteinatlas.org). Expression levels of NPM1, RAD50 and XRCC5 proteins were analyzed in 11, 9 and 6 tumors respectively, derived from high grade serous adenocarcinoma patients (HPA accession nos. ENSG00000181163, ENSG00000113522 and ENSG00000079246 respectively). The red, yellow and light brown colour boxes showing strong, moderate and weak expression (antibody staining intensity) of these targets in the HPA database, while white box represents negative expression. The length of boxes in ovarian cancer indicates overall cancer tissue staining statistics in percentage. Data is indicated as mean ± SE of triplicate experiments. *p <0.05,**p <0.01.

An earlier study on proteomic profiling of the SeOvCa progression in our lab had categorized differentially identified proteins into two groups, where *Group I* comprised EEx and LEx sub-groups consisting 10 and 34 proteins respectively, while *Group II* comprised 31 and 48 differentially upregulated proteins identified in A4-P and A4-T cells respectively (Figure 1A). Identification of 34 proteins in transformed A4 cells (LEx proteins in *Group I*) led to the derivation of 14 diverse cellular processes based on literature based functional annotation (Additional file 2). Towards understanding the functional relevance of LEx proteins, we focused on NPM1 that participates in six of the above pathways. Very limited information is available on its role in DSB repair, wherein two other proteins *viz.* RAD50 and XRCC5 were also identified as being LEx proteins. We thus decided to explore the functional relevance of these proteins in the transformed cells since aberrations in this pathway contribute to genetic (in)stability in several malignancies. As an initial step, we profiled RAD50, NPM1 and XRCC5 protein expression, which confirmed their enriched levels in the transformed or A4-T cells (Figure 1B), though much lower levels can be detected in A4-P cells. This was more striking for NPM1 than the other two proteins (Figure 1C). Expression profiling in HPA (Human protein atlas) revealed enrichment of these in ovarian cancer samples and cell line, though lower levels in stromal and follicle cells were also marked (Figure 1D). Expression levels of these DSB-repair proteins were further profiled in various cancer and leukemic cell lines expression data available in the HPA database. All three proteins were enriched in various cancer cell lines, while levels of NPM1 and XRCC5 were more significantly elevated in leukemic cell lines (Additional file 3A,B,C).

**Figure 2:**
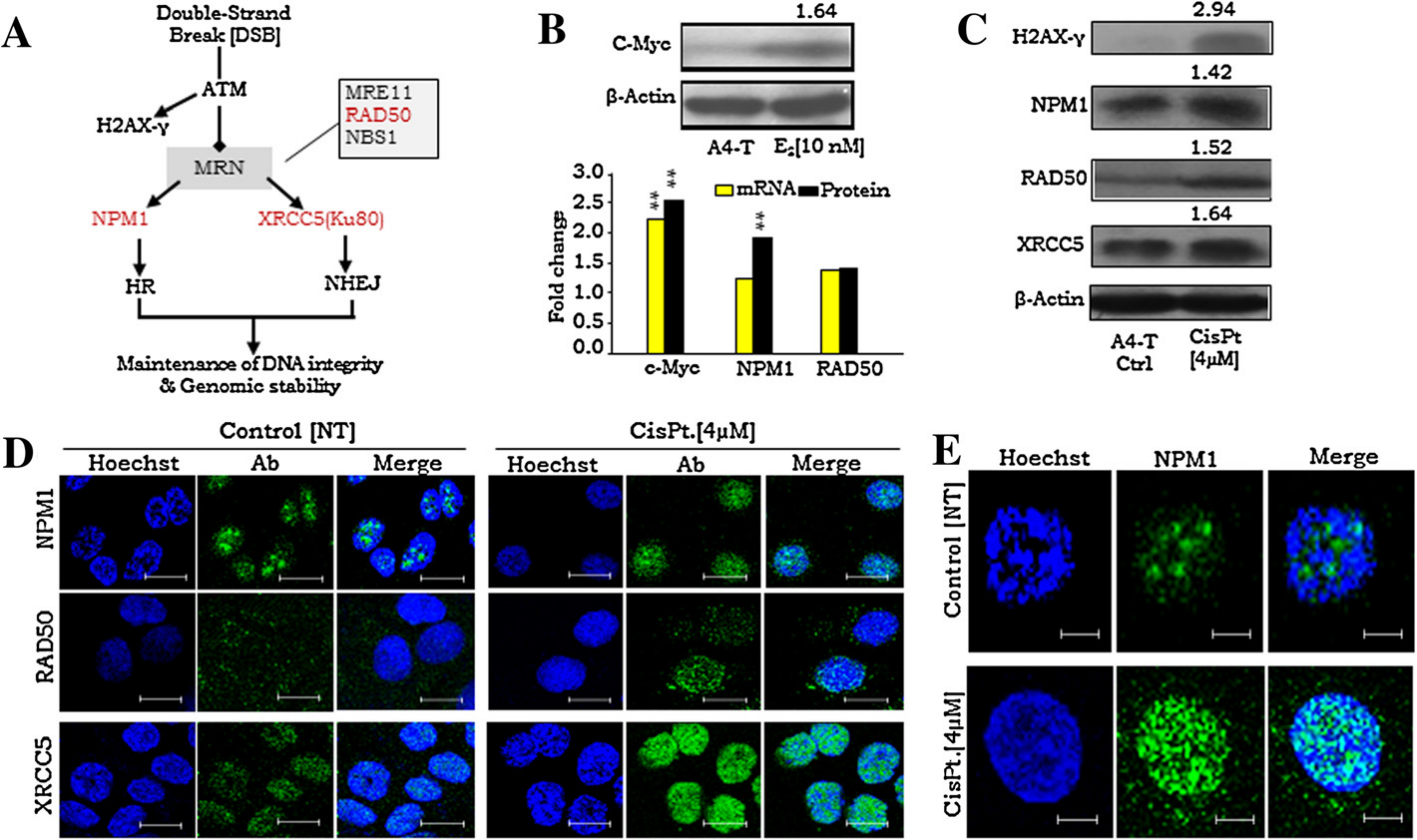
**Status of RAD50, NPM1 and XRCC5 in DSB-repair pathway and modulation of their expression on E**_**2 **_**and cisplatin treatment. A**. Schematic diagram showing designations of RAD50, NPM1 and XRCC5 in HR and NHEJ pathways of DSB-repair. **B**. Representative immunoblot showing elevated expression of c-Myc on 10nM E_2_ treatment (upper panel); fold-change expression of c-Myc, NPM1 and RAD50 mRNA and protein (lower panel). **C**. Representative immunoblots of H2AX-γ, NPM1, RAD50 and XRCC5 on 4 μM Cisplatin treatment; numbers indicate mean fold-change values. **D**. Immunofluorescence staining indicating NPM1, RAD50 and XRCC5 expression on 4 μM Cisplatin treatment in comparison to untreated control A4-T cells; Scale bar 25 μm. **E**. Immunofluorescence staining showing translocation of NPM1 from nucleolus to nucleoplasm within 24 h upon 4 μM cisplatin treatment in A4-T cells in comparison to untreated cells. Scale bar 8 μm. Data shown as mean ± SE of triplicate experiments. *p <0.05,**p <0.01.

In DSB-repair pathway, RAD50 serves as a key component of MRN complex which is regulated by ATM kinase signaling and bilaterally involved in DSB repair and thereby regulates both HR and NHEJ pathways; while NPM1 and XRCC5 are associated with HR and NHEJ pathways respectively (Figure 2A) [9-15]. c-Myc as being a transcription factor, is known to modulate process of cellular transformation. Our earlier studies suggest a role for c-myc in transformation in HGSC [20]. Towards examining its role in SeOvCa progression model, total identified proteins in A4-T cells were matched with online c-Myc portal (http://www.myc-cancer-gene.org). This identified 3 and 8 proteins as transcriptional targets of c-Myc in *Group I and II* respectively (Additional file 4A). Most of these targets were found to be associated with protein metabolism, cell redox reactions and DSB-repair pathways (Additional file 4B). NPM1 and RAD50 were thus suggested to be transcriptional targets of c-Myc [21]; while c-Myc itself is known to be regulated at the transcriptional level by estrogen through the estrogen receptor [22,23]. We have earlier reported ER-α expression in A4 cells [20]; thus, we tested and validated the functionality of such possible cross-talk by treatment of A4-T cells with 10nM E_2_ that consecutively led to elevated c-Myc levels (Figure 2B – upper panel; Additional file 4C) followed by enhanced NPM1 expression (Figure 2B – lower panel) suggesting a regulatory association of c-Myc in transcriptional regulation of NPM1. However, such a correlation with RAD50 expression could not be seen (Figure 2B – lower panel).

### Transformation-associated DSB-repair proteins are responsive to genotoxic stress

Towards investigating the relevance of DSB-repair on genotoxic stress, we induced DSB with 4 μM cisplatin-a genotoxic agent known to form interstrand and intrastrand adducts upon interaction with DNA [24]. The expression of DSB marker i.e. H2AX-γ was significantly upregulated along with the levels of all three DSB repair molecules, though it was much marked for RAD50 and XRCC5 (Figure 2C, 2D). An interesting feature observed was that while the levels of NPM1 were drastically increased on transformation, they were marginally altered on subsequent exposure to genotoxic stress. On profiling its localization at steady-state and under DSB stress, NPM1 was seen to be exported from the nucleolus to the nucleoplasm within 24 h of cisplatin treatment (Figure 2E). The cells treated with cisplatin showed surface granulations and condensed nuclei in comparison to untreated control that indicated onset of cellular stress and apoptosis in a significant cellular fraction (Additional file 4D,E,F). The translocation of NPM1 from the nucleolus to the nucleoplasm may suggest activation and a shift to perform its functional role in DSB-repair.

### Impaired DSB-repair and genotoxic stress leads to cellular apoptosis

**Figure 3:**
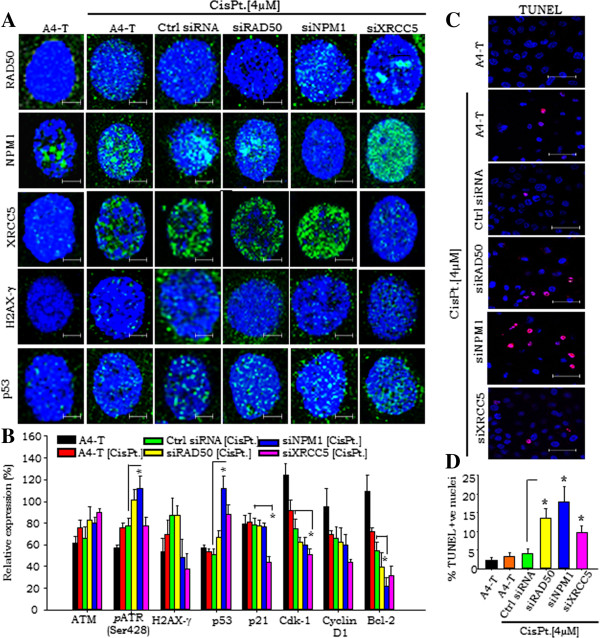
**DSB response triggers p53 signaling under genomic stress. A**. Immunofluorescence staining showing expression of RAD50, NPM1, XRCC5, H2AX-γ and p53 at steady state and 4 μM cisplatin treated control siRNA, siRAD50, siNPM1 and siXRCC5 A4-T cells. **B**. Quantitation of ATM, H2AX-γ, p53, p21, CDK1, Cyclin D1 and Bcl-2 protein expressions in untreated and cisplatin treated A4-T, control siRNA, siRAD50, siNPM1 and siXRCC5 A4-T cells. **C**. *In-Situ* fluorescein cell death (TUNEL) analysis showing nuclear fragmentation (red foci) in untreated and cisplatin treated control siRNA, siRAD50, siNPM1 and siXRCC5 A4-T cells (Red color- pseudo color instead of green). **D**. Quantitative analysis of TUNEL assay showing percentage apoptotic populations in complete set of untreated and cisplatin treated A4-T, control siRNA, siRAD50, siNPM1 and siXRCC5 cells. Scale bar is 12 μm. Data shown as mean ± SE of triplicate experiments. *p <0.05; **p <0.01; ***p <0.001.

In order to elucidate the functional role of these proteins in DSB repair, we silenced each of these three key molecules in A4-T cells followed by 4 μM cisplatin treatment. A significantly higher XRCC5 levels in NPM1 silenced cells and *vice-versa,* was observed (Additional file 5A, B). Cisplatin treatment led to an increment in H2AX-γ, RAD50, NPM1, XRCC5 and p53 in all treated sets. Numbers of p53 nuclear foci in DSB-repair protein silenced cells observed higher in comparison to the siRNA control cells (Figure 3A). Silencing of RAD50 under genotoxic stress increased the number of p53 foci in treated cells that may correlate with functionally compromised MRN complex; though this increment in the p53 foci in siNPM1 cells appeared higher in comparison to the siRAD50 silenced cells. Increased p53 foci in NPM1 silenced cells suggest defective HR pathway and also loss of NPM1 regulation of p53 activity [25], while those in XRCC5 silenced cells may predict fate of cells with impaired NHEJ pathway. Further, high frequency of p53 nuclear foci in siNPM1 and siXRCC5 cells, and a relatively lower one in siRAD50 cells was observed (Figure 3B, Additional file 5C).

NPM1 silencing led to significantly elevated XRCC5 levels in these compromised HR DSB-repair cells; likewise, NHEJ compromised cells on XRCC5 silencing exhibited elevated NPM1 levels (Additional file 5B). Such enhanced expression of XRCC5 and NPM1 across alternatively silenced cells indicated prompt activation of respective NHEJ or HR pathways in these impaired cells. In sensitive cells, cisplatin treatment is known to regulate p53 stability and transcriptional activity upon activation of ATM and ATR kinase, while the transient sub-nuclear redistribution of NPM1 may suggest its activation through a direct interaction with p53 independently of ARF [10,25]. The suggested regulatory cross-talk with p53 prompted us to further profile the protein expression of some key p53-regulatory network molecules activated by ATR phosphorylation (Ser428) or ATM and H2AX-γ expression in cisplatin treated cells (Figure 3B, Additional file 5D). The activated p53 with its down-stream modulator p21 suggested lower Bcl-2 levels in NPM1 silenced cells in comparison to control siRNA transfected cells, though p21 levels appear unaltered throughout the set that may reflect transient stability or a sort half-life of p21 protein. A slightly higher Bcl-2 level in siXRCC5 cells in comparison to siNPM1 and siRAD50 cells may suggest stimulation of the error-free DSB-repair mediated through HR pathway, which may further rescue cells from p53-mediated apoptosis. Lower levels of Cyclin D1 and Cdk1 in DSB-repair protein silenced cells in comparison to control siRNA cells exhibits p53/p21 mediated control over cell cycle progression in RAD50, NPM1 and XRCC5 silenced cells, though individual Cdk-1 levels in siXRCC5 cells were found significantly lower. We observed incidences of nuclear fragmentation in RAD50 and NPM1 silenced cells in comparison to control siRNA transfected cells (Additional file 5E). The consequence of these incidences i.e. cellular apoptosis were quantified through TUNEL assay in the complete treatment set (Figure 3C) wherein, higher levels of nuclear fragmentation in the RAD50 and NPM1 silenced cells in comparison to control siRNA transfected cells corroborate with the above results (Figure 3D).

### Compromised HR & NHEJ-repair pathways result in genomic instability in transformed cells

**Figure 4:**
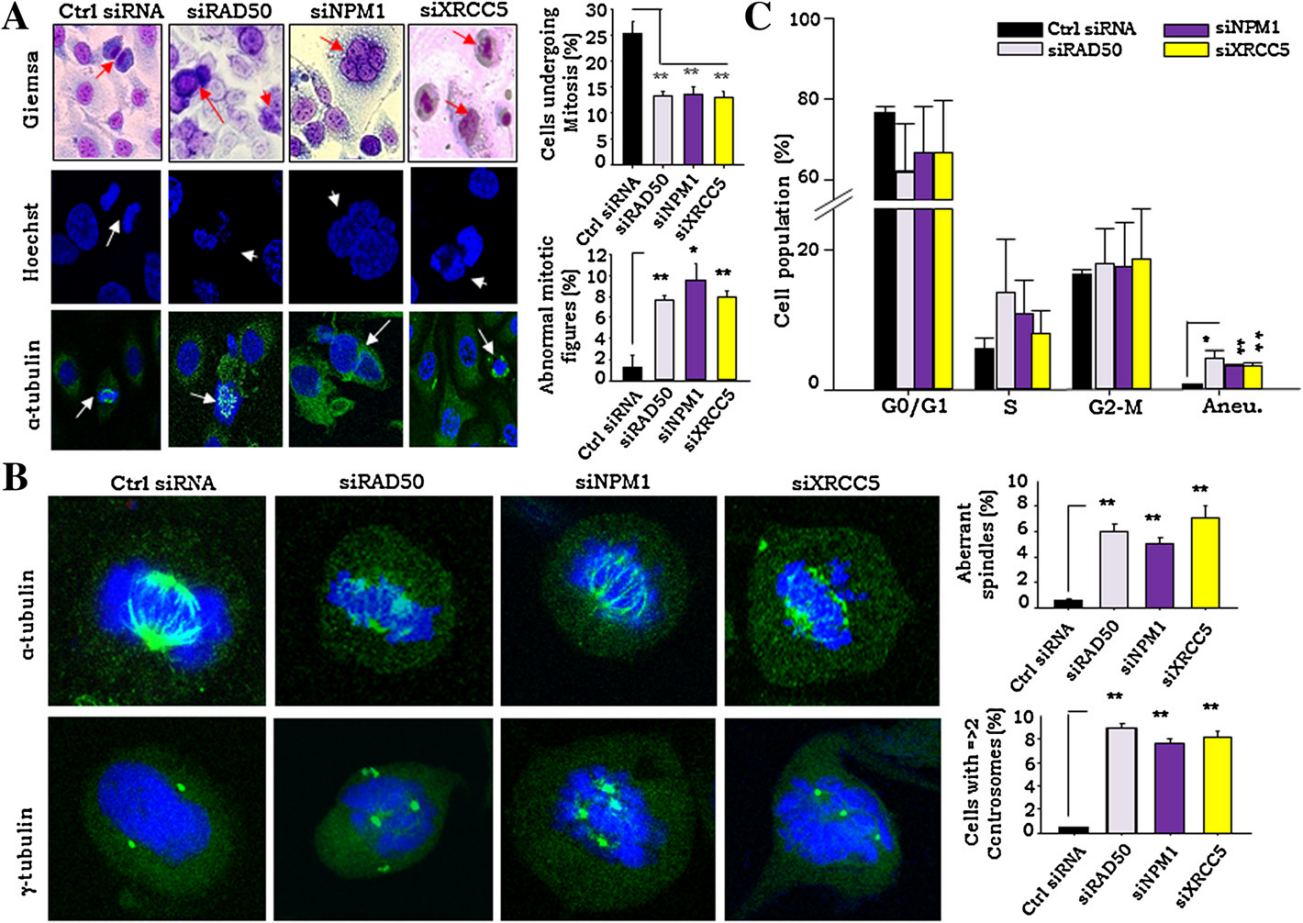
**Ablation of RAD50, NPM1 and XRCC5 lead to genomic instability and facilitate multinucleated cells and mitotic irregularities. A**. Representative Images of control siRNA, siNPM1, siRAD50 and XRCC5 A4-T cells - panels indicate Giemsa (top), Hoechst (middle) and α-tubulin stained (lower) nuclei respectively. Marked arrow indicating incidences of abnormal mitotic figures, multi-nucleation and mitotic irregularities in the siRAD50, siNPM1 and siXRCC5 cells respectively, while in control siRNA transfected cells it is denoting normal mitosis; 40X magnification; 50 μm scale bar. Representative graphs indicating frequencies of mitosis and abnormal mitotic figures in control and siRAD50, siNPM1 and siXRCC5 cells. **B**. Representative images of cells stained for α- and γ- tubulin expression indicating existence of abnormal spindles as well as numbers and position of centrosomes during mitosis in control and siRAD50, siNPM1 and siXRCC5 cells; representative graphs indicating frequencies of aberrant spindle formation and cells with more than 2 centrosomes in control and siRAD50, siNPM1 and siXRCC5 cells on the right. **C**. Graphical representation of flow cytometry based resolution of cell cycle phases and aneuploid genomic content associated with indicative of control and siRAD50, siNPM1 and siXRCC5 cells through PI staining. Data shown as mean ± SE of triplicate experiments. *p <0.05; **p <0.01.

The above results suggest that cells with compromised DSB-repair on being subjected to stress are frequently eliminated through p53/p21 led apoptosis at a high frequency. We further probed the effects of defective HR & NHEJ pathways on mitosis and genomic integrity in the cells surviving under the same stress through a detailed screening of nuclear morphology. A close investigation of nuclear morphology in RAD50, NPM1 and XRCC5 silenced cells showed frequent incidences of improper nuclear segregation (legging chromosome), multi-nucleated cells and abnormal mitotic figures (Figure 4A; Additional file 6A). This consistently revealed 7–9 fold higher nuclear irregularities of multi-nucleation, abnormal and reduced mitoses in compromised DSB-repair cells in comparison to the siRNA control (Figure 4A). A detailed analysis of nuclear morphology at steady state and mitosis evidently revealed occurrence of nuclear condensation and faulty mitotic progression in RAD50, NPM1 and XRCC5 silenced cells (Additional file 6B).

We further analyzed mitotic nuclei to demonstrate the probability of aberrant spindle formation in DSB-repair deficient cells. The α-tubulin and γ-tubulin staining of mitotic nuclei revealed 5–9 fold aberrant mitotic-spindle formation and higher numbers (>2) of centrosomes in DSB-repair deficient cells (Figure 4B). Cyclin E-Cdk2 mediated phosphorylation of NPM1 has been suggested to dissociate its binding from centrosomes at the G1-phase of the cell cycle and permit centrosome duplication prior to cell division [26]. NPM1 silencing thereby could lead to unrestricted centrosome duplication and impaired cell division followed by multinucleation. On the other hand, RAD50 and XRCC5 silencing led to irregular chromosomal segregation generating abnormal mitotic figures. Such association of mitotic irregularities with impaired DSB repair led us to examine genomic content and ploidy levels in the silenced cells under stress. While the former was not significantly altered, the incidence of aneuploidy was enhanced 5–7 fold times in cells with compromised DSB-repair in comparison to the transfected siRNA controls (Figure 4C). Occurrence of mitotic irregularities and analyses of genomic content in RAD50, NPM1 and XRCC5 silenced cells demonstrated gain of genomic instability. Further, MAD2 and BUBR1 levels (upregulations of which are known to be associated with genomic instability) were studied under genotoxic-stress (Additional file 6C). Expressions of MAD2 were found higher in RAD50 and NPM1 silenced cells, while BUBR1 levels were slight high in RAD50 silenced cells. Elevated expression of MAD2 in these silenced cells may suggest progression of genomic instability. Together, the data identifies defective DSB-repair driven genomic instability (GI) that could lead to altered ploidy levels in transformed cells under genomic-stress.

## Discussion

**Figure 5:**
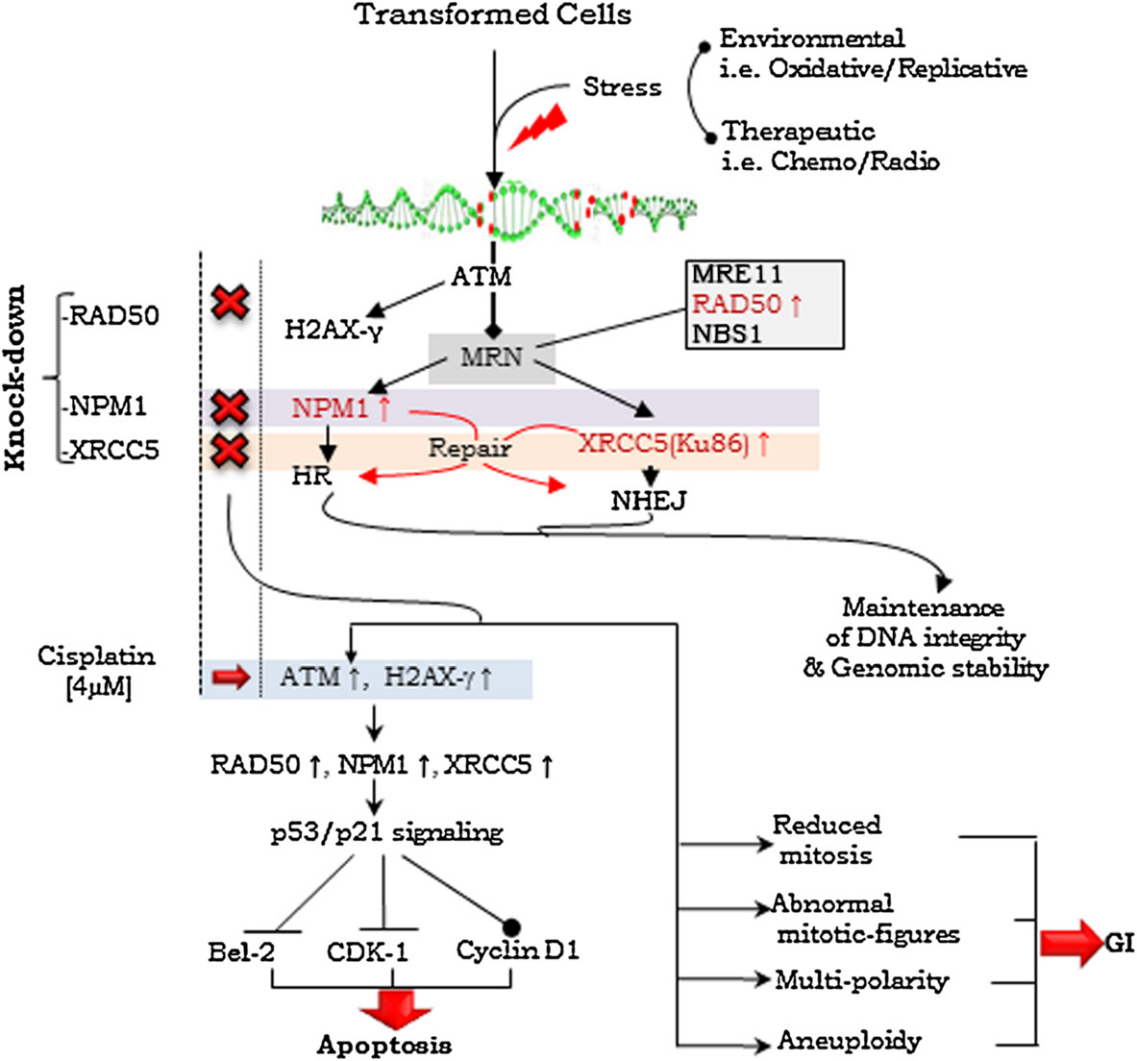
**Schematic model demonstrating functional involvement of NPM1, RAD50 and XRCC5 in DSB-repair in the progression of ovarian cancer cells.** Onset of environmental and therapeutic stress leads to the activation of ATM, which recruits MRN complex and phosphorylates H2AX histone variant. NPM1 and XRCC5 involved in HR and NHEJ pathways respectively. Knock-down of NPM1 and XRCC5 across alternatively activates NHEJ and HR pathways (red lines) towards maintaining DNA integrity and genomic stability. Under genomic stress conditions, knock-down of RAD50, NPM1 and XRCC5 DSB-repair proteins lead to the activation of p53/p21 signaling pathway resulted in onset of apoptosis of DSB-repair impaired cells. On other hand, cells surviving with impaired DSB-repair exhibit frequencies of reduced mitosis, mitotic irregularities and multi-polarity and aneuploidy, which conclusively facilitate genomic instability in transformed cells.

In the present study, we investigated the functional relevance of RAD50, NPM1 and XRCC5 in HR and NHEJ pathways and explored their vital role in the process of DSB-repair and evasion of apoptosis (Figure 5). Silencing of the molecular expression of these key proteins leads to aberrant functioning of DSB-repair pathways. It has been shown earlier that loss of NPM1 expression could elevate levels of H2AX phosphorylation on exposure to UV radiation or DNA damage. In the HR DSB-repair pathway, recruitment of phosphorylated NPM1 by ubiquitin conjugates downstream of RNF8 and RNF168 has been suggested [11]. Differentially upregulated levels of two other DSB molecules *viz.* RUVBL1 and RUVBL2 was also affirmed in A4-T cells, wherein RUVBL1 is known to be associated with c-Myc in promoting cellular transformation [21]. In the present study, c-Myc mediated modulation of NPM1 levels in an E_2_ dependent manner was affirmed that indicates responsiveness of c-Myc to E_2_. Together, this suggests the subsistence of several pathways that cross-talk with each other.

Induction of H2AX-γ in response to genotoxic-stress provided a suitable system to investigate the process of DSB-repair in transformed cells. A nucleolar-nuclear translocation of NPM1 on cisplatin induced genotoxic-stress comprising a swift DSB-repair response in the nucleoplasm was identified. High grade SeOvCa has been suggested to harbor p53 mutation at advanced stage [27], though it is found limited to 91-96% in serous malignancies [3,28,29]. Activation of ATM dependent p53/p21 signaling in compromised DSB repair cells leads to downstream control over Bcl-2, Cdk-1 and Cyclin D1 expression, such mechanistically coordinated process affirms active p53 status in A4-T cells. Presence of stable p21 levels, despite p53 activation justifies transient expression of p21 protein upon DNA damage. RAD50 silencing apparently limits process of DSB repair down-stream of the MRN complex; while activation of NHEJ or HR pathways in NPM1 or XRCC5 silenced cells interestingly demonstrates alternative strategies for DSB-damage repair (Figure 5). Higher frequency of apoptosis in NPM1 silenced cells in comparison to RAD50 and XRCC5 silenced may justify consequence of cumulative shut-down of several functions mediated by NPM1 [25,26]. Compromised DSB-repair process leads to the mitotic irregularities and multinucleation; wherein unrestricted centrosome duplication in the absence of NPM1 has been reported [26]. Defective repair in NPM1 silenced cells indicates probability of error-prone chromosomal segregation of unrepaired DNA, resulted in generation of aberrant and mutipolar mitotic spindles. Our findings suggest enhanced expression of these three DSB repair molecules is a crucial event during SeOvCa transformation in order to maintain DNA integrity and genomic stability (Figure 5). Conclusively, we demonstrated that silencing of NPM1, RAD50 and XRCC5 in transformed cells led to mitotic irregularities and aneuploidy. Elevated levels of these DSB-markers in transformed cells are essential to compensate the effects of high risk of DSB events in transformed cells. In present study, analysis of the functional relevance of these proteins of DSB-repair extends current understanding of the process of cellular transformation during progression of SeOvCa.

## Abbreviations

EOC: Epithelial ovarian cancer; SeOvCa: Serous ovarian adenocarcinoma; DSB: Double-strand break; HR: Homologous recombination; NHEJ: Non-homologous end joining; DDR: DNA damage repair; NPM1: Nucleophosmin; RAD50: Human homolog of *Saccharomyces cerevisiae* Rad50; XRCC5: X-ray repair cross-complementing 5; GI: Genomic instability.

## Competing interests

The authors declare that they have no competing interests.

## Authors’ contributions

RSK performed the experiments and prepared the manuscript. SAB conceived the study, participated in its design and coordination, corrected the manuscript and supervised the project. Both authors read and approved the final manuscript.

## Supplementary Material

Additional file 1 Derivation of A4 clone and establishment of A4 SeOvCa progression model. Click here for file

Additional file 2 **Functional categorization of identified LEx proteins.** Table showing functional annotation based categorized of 34 LEx proteins to 14 cellular processes in transformed SeOvCa cells. Click here for file

Additional file 3 **NPM1, RAD50 and XRCC5 levels in HPA database.** Expression profiling of NPM1 (A), RAD50 (B) and XRCC5 (C) in cancer and leukemic cell lines analyzed in the Human Protein Atlas (HPA) database (http://www.proteinatlas.org). The red, yellow and light brown colour boxes showing strong, moderate and weak expression (antibody staining intensity) of these targets in the HPA database. The horizontal dotted line represents mean of individual protein expression in various human cell lines and leukemia samples, wherein dashed line represents range of upper and lower expression values. Mean expression of the individual proteins were represented in percentage with reference to the internal immunostaining control. Click here for file

Additional file 4 **Identification of c-Myc targets in DSB-repair functional group and E**_**2 **_**and cisplatin mediated expression modulation.** A. List of suggested c-Myc targets identified in qualitative and differential upregulated groups of A4 transformed cells. B. Diagram showing major cellular processes, categorized based on functional annotation of identified c-Myc gene targets in A4 transformed cells. C. Quantitation of c-Myc expression in untreated [NT] control and Estradiol [E_2_] treated A4 transformed cells validated by immunoblotting; representative graph shows c-Myc expressions relative to the β-actin (represented in percentage post normalization with β-actin). D. Cisplatin treatment induced cellular stress, where panel showing untreated (a) and drug treated (b) A4 transformed cells. E. FACS profiles showing comparative cisplatin mediated apoptosis in untreated and cisplatin treated A4 transformed cells. F. Quantitation of apoptosis. Error bars represent S.E. (n = 3). Data were shown as means ± SE of triplicate experiments. *p <0.05 and **p <0.01. Click here for file

Additional file 5 **Evaluation of p53/p21 pathways activation in NPM1, RAD50 and XRCC5 silenced cells under genetoxic stress.** A. Representative immunoblots of NPM1, RAD50 and XRCC5 in control siRNA, siNPM1, siRAD50 and siXRCC5 silenced cells. B. Quantitation of NPM1, RAD50 and XRCC5 protein expression in control siRNA and siNPM1, siRAD50 & siXRCC5 transfected cells in cisplatin treated cells; relative expression of these proteins were calculated in percentage upon normalization with β-actin. C. Quantitative analysis of p53 foci in the nucleus of cisplatin treated control siRNA and siNPM1, siRAD50 & siXRCC5 transfected cells. D. Representative immunoblots of ATM, *p*ATR (Ser428), H2AX-γ, RAD50, NPM1, XRCC5, p53, p21, CDK1, Cyclin D1, Bcl-2, Exo1 and β-actin expressions in untreated and cisplatin treated A4-T, control siRNA, siNPM1, siRAD50 and XRCC5 silenced A4 transformed cells. E. Immunofluorescence staining showing nuclear fragmentation in NPM1 and RAD50 silenced cells on genotoxic stress. Scale bar is 37.5 μm. Click here for file

Additional file 6 **NPM1, RAD50 and XRCC5 deficiency led to mitotic irregularities followed by genomic instability.** A. Immunofluorescence panel showing mitotic irregularities in RAD50, XRCC5 silenced cells, wherein NPM1 silenced cells showing multinucleation in comparison to the normal mitosis in control siRNA (indicated by white arrow in control and RAD50, NPM1 and XRRC5 silenced cells); B. Representative Giemsa stained images of untreated A4-T (i) and 4 μM cisplatin treated control siRNA, siNPM1, siRAD50 and siXRCC5 A4-T cells (panels ii, iii, iv, v, vi respectively) in two sets; upper images showing steady state while lower’s are mitotically active A4 transformed cells (black arrows in siNPM1, siRAD50 and siXRCC5 cells indicating mitotic irregularities, while in cisplatin treated control cells and control siRNA treated cells its showing condense nuclear configures). Scale bar is 50 μm. Focus 40X. C. Immunofluorescence staining showing expressions of MAD2 and BUBR1 in untreated and cisplatin treated A4-T, control siRNA, RAD50, NPM1and XRCC5 silenced A4 transformed cells. Click here for file
