# Supplementary material for: Enhanced levels of double-strand DNA break repair proteins protect ovarian cancer cells against genotoxic stress-induced apoptosis
Source: J Ovarian Res. 2013 Sep 17;6:66. doi: 10.1186/1757-2215-6-66 (PMC3848582; doi:10.1186/1757-2215-6-66)
Supplement: Additional file 1 — Derivation of A4 clone and establishment of A4 SeOvCa progression model. [file 1757-2215-6-66-S1.pdf]

Additional file 1: Establishment of epithelial ovarian transformation model system

In 2005, an *in vitro* progression model of serous ovarian adenocarcinoma (SeOvCa) transformation was established in our lab (Bapat *et al.* 2005). Briefly, one of the 19 spontaneously immortalized single cell clones isolated from the malignant multi-layered ascites of the grade IV SeOvCa patient developed into a progression model system of immortal (i) pre-transformed (A4-P) cells showing slow-cycling property and inability to produce xenograft in immunocompromised mice, and (ii) transformed A4 (A4-T) cells, which demonstrated aggressive, metastatic and tumorigenic capacity. Further, clonogenic and spheroid generation assays confirmed aggressiveness and growth potential of A4-T cells. Establishment of SeOvCa progression model provided a suitable *in vitro* system to profile altered molecular expression of proteins associated with transformation process that are indicative of the mechanisms underlying development of malignancy and may also have implications in prevention of disease progression.
