# Supplementary material for: Enhanced levels of double-strand DNA break repair proteins protect ovarian cancer cells against genotoxic stress-induced apoptosis
Source: J Ovarian Res. 2013 Sep 17;6:66. doi: 10.1186/1757-2215-6-66 (PMC3848582; doi:10.1186/1757-2215-6-66)
Supplement: Additional file 2 — Functional categorization of identified LEx proteins. Table showing functional annotation based categorized of 34 LEx proteins to 14 cellular processes in transformed SeOvCa cells. [file 1757-2215-6-66-S2.pdf]

**Additional file.2:** Functional annotation based categorized of 34 LEx proteins to 14 cellular processes in transformed SeOvCa cells.

| S.No. | Functional group/Cellular process | Categorized proteins |
|---|---|---|
| 1 | Chemoresistance | HSPD1, PRDX2, PDIA3, SOD1 |
| 2 | Resistance to Apoptosis | PRDX2, PDIA3, SOD1, NPM1 |
| 3 | Oxidative stress | SOD1, NPM1, PRDX2, RNASEL, ENO2 |
| 4 | Transcriptional regulation and Maintenance | MAGOH, XRCC5, RAD50, CBX5, NPM1 |
| 5 | Invasion and Proliferation | NPM1, PRDX2 |
| 6 | Transport and Signaling | CLIC4, SSR4, KPNA4 |
| 7 | RNA Metabolism | SNRPF, NPM1 |
| 8 | DSB Repair | NPM1, RAD50, XRCC5 |
| 9 | Protein Synthesis | EEF1A1, TUFM |
| 10 | Protein Degradation | PSMA1, PSMB3, PSMC2, PSMC4 |
| 11 | Protein Folding and Isomerization | HSPD1, PDIA3, CCT3, CCT8, FKBP4 |
| 12 | Energy Production and Metabolism | ATP5B, ATP5D, ENO2, ECH1, PKM2 |
| 13 | Immunological Responses | B2M, SCRN |
| 14 | Structural and secretory proteins | MYO1D, ALB |
