# Supplementary figures and images for: Enhanced levels of double-strand DNA break repair proteins protect ovarian cancer cells against genotoxic stress-induced apoptosis
Source: J Ovarian Res. 2013 Sep 17;6:66. doi: 10.1186/1757-2215-6-66 (PMC3848582; doi:10.1186/1757-2215-6-66)

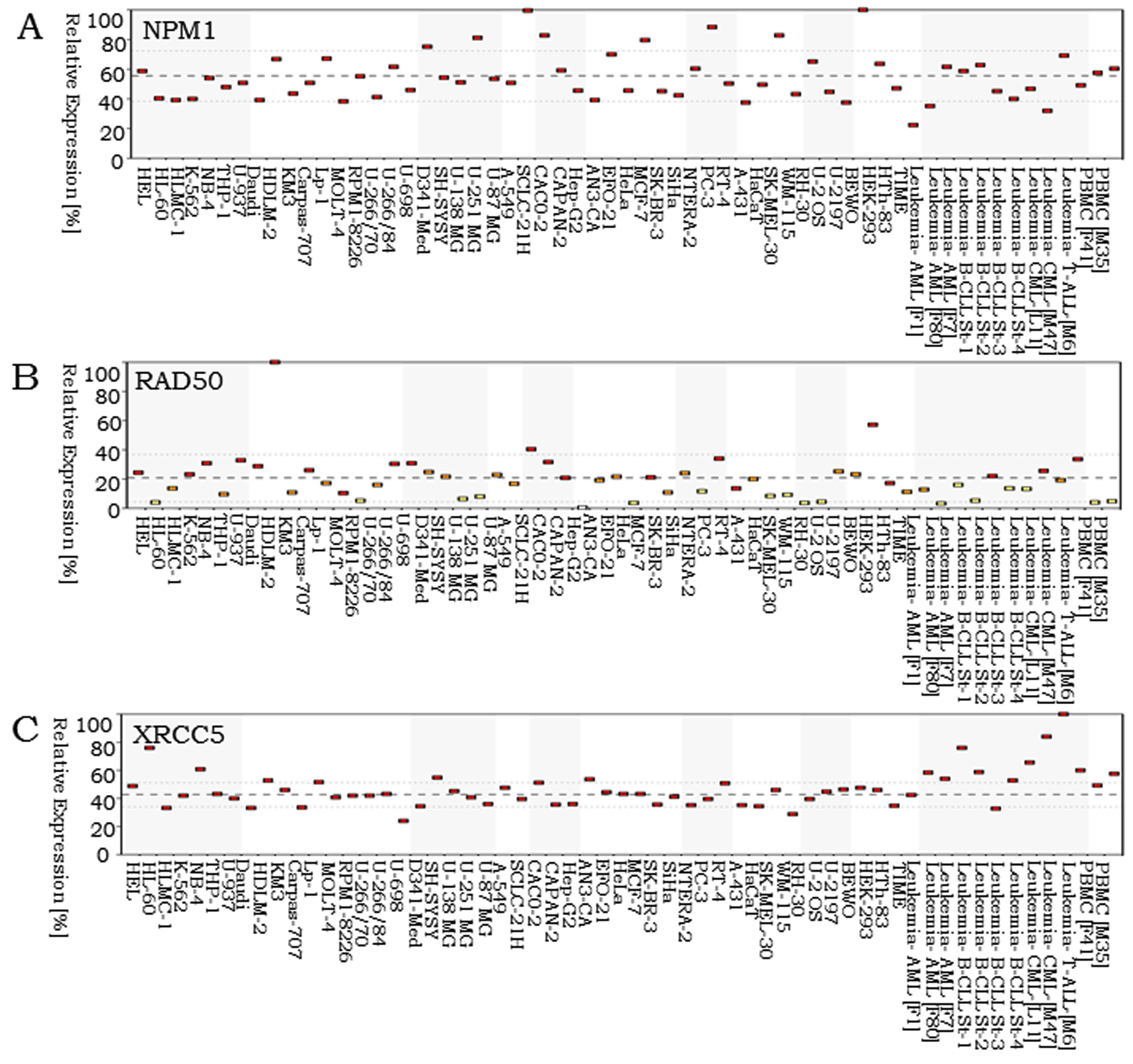

Supplement: Additional file 3 — NPM1, RAD50 and XRCC5 levels in HPA database. Expression profiling of NPM1 (A), RAD50 (B) and XRCC5 (C) in cancer and leukemic cell lines analyzed in the Human Protein Atlas (HPA) database (http://www.proteinatlas.org). The red, yellow and light brown colour boxes showing strong, moderate and weak expression (antibody staining intensity) of these targets in the HPA database. The horizontal dotted line represents mean of individual protein expression in various human cell lines and leukemia samples, wherein dashed line represents range of upper and lower expression values. Mean expression of the individual proteins were represented in percentage with reference to the internal immunostaining control. [file 1757-2215-6-66-S3.tiff]

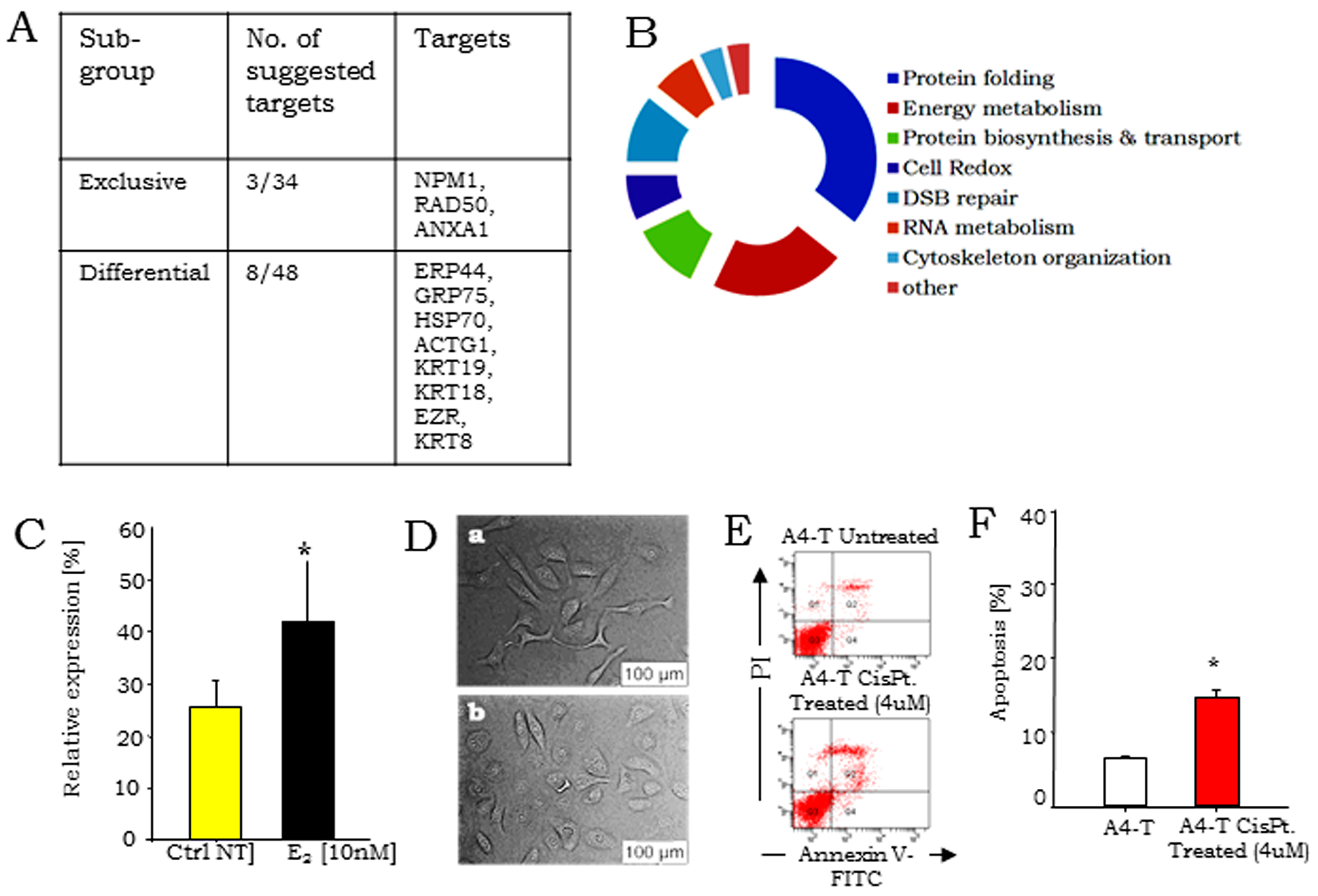

Supplement: Additional file 4 — Identification of c-Myc targets in DSB-repair functional group and E2 and cisplatin mediated expression modulation. A. List of suggested c-Myc targets identified in qualitative and differential upregulated groups of A4 transformed cells. B. Diagram showing major cellular processes, categorized based on functional annotation of identified c-Myc gene targets in A4 transformed cells. C. Quantitation of c-Myc expression in untreated [NT] control and Estradiol [E2] treated A4 transformed cells validated by immunoblotting; representative graph shows c-Myc expressions relative to the β-actin (represented in percentage post normalization with β-actin). D. Cisplatin treatment induced cellular stress, where panel showing untreated (a) and drug treated (b) A4 transformed cells. E. FACS profiles showing comparative cisplatin mediated apoptosis in untreated and cisplatin treated A4 transformed cells. F. Quantitation of apoptosis. Error bars represent S.E. (n = 3). Data were shown as means ± SE of triplicate experiments. *p <0.05 and **p <0.01. [file 1757-2215-6-66-S4.tiff]

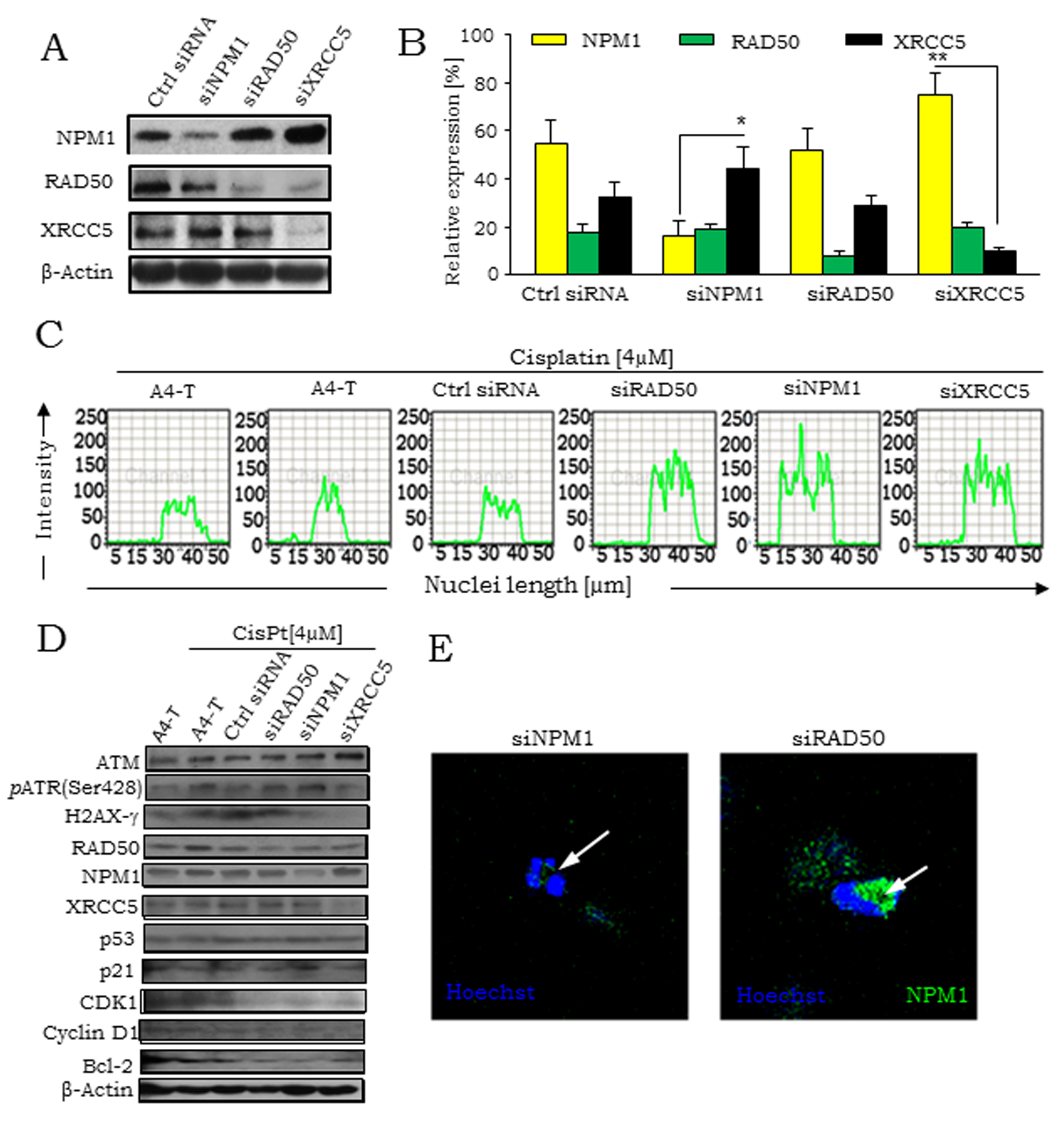

Supplement: Additional file 5 — Evaluation of p53/p21 pathways activation in NPM1, RAD50 and XRCC5 silenced cells under genetoxic stress. A. Representative immunoblots of NPM1, RAD50 and XRCC5 in control siRNA, siNPM1, siRAD50 and siXRCC5 silenced cells. B. Quantitation of NPM1, RAD50 and XRCC5 protein expression in control siRNA and siNPM1, siRAD50 & siXRCC5 transfected cells in cisplatin treated cells; relative expression of these proteins were calculated in percentage upon normalization with β-actin. C. Quantitative analysis of p53 foci in the nucleus of cisplatin treated control siRNA and siNPM1, siRAD50 & siXRCC5 transfected cells. D. Representative immunoblots of ATM, pATR (Ser428), H2AX-γ, RAD50, NPM1, XRCC5, p53, p21, CDK1, Cyclin D1, Bcl-2, Exo1 and β-actin expressions in untreated and cisplatin treated A4-T, control siRNA, siNPM1, siRAD50 and XRCC5 silenced A4 transformed cells. E. Immunofluorescence staining showing nuclear fragmentation in NPM1 and RAD50 silenced cells on genotoxic stress. Scale bar is 37.5 μm. [file 1757-2215-6-66-S5.tiff]

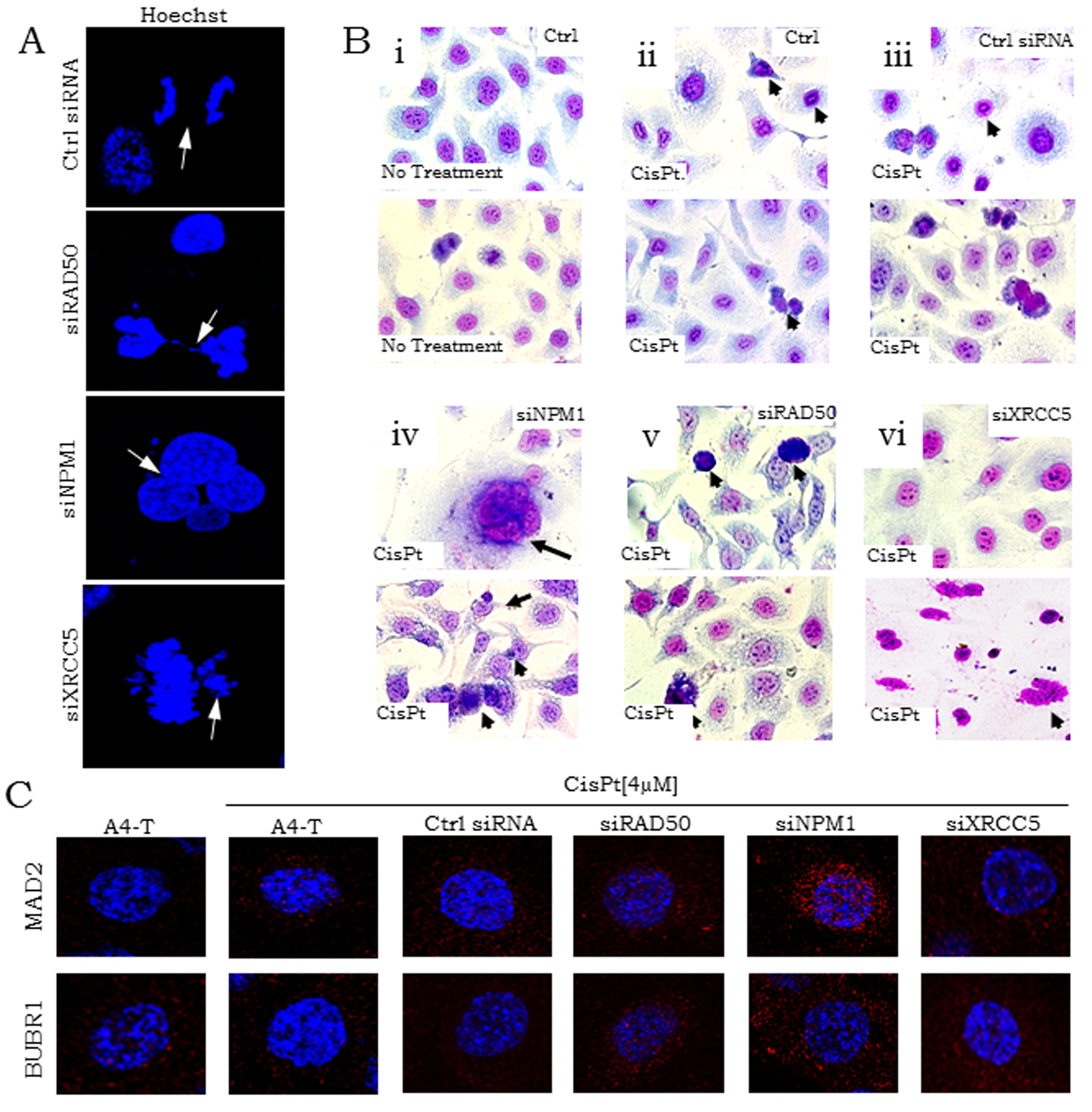

Supplement: Additional file 6 — NPM1, RAD50 and XRCC5 deficiency led to mitotic irregularities followed by genomic instability. A. Immunofluorescence panel showing mitotic irregularities in RAD50, XRCC5 silenced cells, wherein NPM1 silenced cells showing multinucleation in comparison to the normal mitosis in control siRNA (indicated by white arrow in control and RAD50, NPM1 and XRRC5 silenced cells); B. Representative Giemsa stained images of untreated A4-T (i) and 4 μM cisplatin treated control siRNA, siNPM1, siRAD50 and siXRCC5 A4-T cells (panels ii, iii, iv, v, vi respectively) in two sets; upper images showing steady state while lower’s are mitotically active A4 transformed cells (black arrows in siNPM1, siRAD50 and siXRCC5 cells indicating mitotic irregularities, while in cisplatin treated control cells and control siRNA treated cells its showing condense nuclear configures). Scale bar is 50 μm. Focus 40X. C. Immunofluorescence staining showing expressions of MAD2 and BUBR1 in untreated and cisplatin treated A4-T, control siRNA, RAD50, NPM1and XRCC5 silenced A4 transformed cells. [file 1757-2215-6-66-S6.tiff]
